# Plasticity of GluN1 at Ventral Hippocampal Synapses in the Infralimbic Cortex

**DOI:** 10.3389/fnsyn.2021.695964

**Published:** 2021-07-15

**Authors:** Yesenia Castillo-Ocampo, María Colón, Anixa Hernández, Pablo Lopez, Yamil Gerena, James T. Porter

**Affiliations:** ^1^Department of Basic Sciences, Ponce Research Institute, Ponce Health Sciences University, Ponce, Puerto Rico; ^2^Department of Pharmacology and Toxicology, School of Medicine, University of Puerto Rico, San Juan, Puerto Rico

**Keywords:** NMDA receptor, GluN1, fear conditioning, rat, synaptosomes, sex difference, synaptic plasticity, channelrhodopsin

## Abstract

Although the infralimbic cortex (IL) is not thought to play a role in fear acquisition, recent experiments found evidence that synaptic plasticity is occurring at ventral hippocampal (vHPC) synapses in IL during auditory fear acquisition as measured by changes in the *N*-methyl-D-aspartate (NMDA) receptor-mediated currents in male rats. These electrophysiological data suggest that fear conditioning changes the expression of NMDA receptors on vHPC-to-IL synapses. To further evaluate the plasticity of NMDA receptors at this specific synapse, we injected AAV particles expressing channelrhodopsin-EYFP into the vHPC of male and female rats to label vHPC projections with EYFP. To test for NMDA receptor changes in vHPC-to-IL synapses after fear learning, we used fluorescence-activated cell sorting (FACS) to quantify synaptosomes isolated from IL tissue punches that were positive for EYFP and the obligatory GluN1 subunit. More EYFP+/GluN1+ synaptosomes with greater average expression of GluN1 were isolated from male rats exposed to auditory fear conditioning (AFC) than those exposed to context and tones only or to contextual fear conditioning (CFC), suggesting that AFC increased NMDA receptor expression in males. In a second experiment, we found that pairing the tones and shocks was required to induce the molecular changes and that fear extinction did not reverse the changes. In contrast, females showed similar levels of EYFP+/GluN1+ synaptosomes in all behavioral groups. These findings suggest that AFC induces synaptic plasticity of NMDA receptors in the vHPC-to-IL projection in males, while female rats rely on different synaptic mechanisms.

## Introduction

Contextual and auditory fear conditioning (AFC) in rodents mimics fear-related behavior in humans (LeDoux, 2000; Phelps and LeDoux, 2005). Using this animal model, researchers identified the interconnected fear circuit with the amygdala as the central structure for fear expression. The intensity of the fear generated by amygdala outputs is attenuated by projections from the infralimbic cortex (IL) (Bloodgood et al., 2018; Bukalo et al., 2021). Abundant evidence suggests that IL plays a central role in fear extinction memory (Chang and Maren, 2010; Milad and Quirk, 2012; Do-Monte et al., 2015; Marek et al., 2018; Tao et al., 2020). Furthermore, contextual and temporal information from the ventral hippocampus (vHPC) determines when IL inhibits fear expression which provides contextual specificity of fear extinction recall (Corcoran et al., 2005; Sierra-Mercado et al., 2011; Marek et al., 2018).

In contrast to the central role of IL in fear extinction, initial studies suggested that fear acquisition did not affect IL and IL activity did not affect fear learning (Milad and Quirk, 2002; Burgos-Robles et al., 2007). However, subsequent research has found evidence that fear acquisition alters the intrinsic excitability of IL neurons (Santini et al., 2008, 2012; Song et al., 2015; Soler-Cedeño et al., 2016; Bloodgood et al., 2018). Furthermore, pharmacologically reducing IL excitability increased fear learning (Santini and Porter, 2010), suggesting that ongoing IL neuronal activity during fear learning was impeding acquisition. Consistent with the rodent studies, recent human studies also found that fear learning alters activity in the ventral medial prefrontal cortex (vmPFC) which is considered to be homologous to the rodent IL (Fullana et al., 2016; Harrison et al., 2017; Dunsmoor et al., 2019) and people with vmPFC lesions show impaired fear acquisition (Battaglia et al., 2020). Taken together these studies suggest that associative fear learning induces plasticity in the homologous structures, the rodent IL, and the human vmPFC.

The acquisition of AFC does not appear to induce widespread synaptic plasticity in IL (Pattwell et al., 2012; Sepulveda-Orengo et al., 2013). In fact, these independent studies found that synaptic plasticity in IL occurs exclusively after fear extinction rather than fear acquisition. However, examination of ventral hippocampal (vHPC) synapses labeled with channelrhodopsin in IL suggests that fear acquisition induces more restricted plasticity at specific synapses in IL (Soler-Cedeño et al., 2019). This study found less *N*-methyl-D-aspartate (NMDA) receptor-mediated current at vHPC-to-IL synapses after AFC in male rats. The finding of less current suggests that AFC reduces the expression of NMDA receptors at vHPC-to-IL synapses in male rats. To address this possibility and determine whether females also show similar synaptic plasticity, we labeled vHPC synapses with AAV expression of channelrhodopsin-EYFP in both sexes and isolated synaptosomes from IL tissue punches after contextual fear conditioning (CFC) and AFC. Since NMDA receptors are composed of two obligatory GluN1 subunits (Paoletti et al., 2013), we decided to quantify GluN1 expression as a relative measure of total NMDA receptor (NMDAR) expression. From the general synaptosome population, vHPC-to-IL synaptosomes were identified and analyzed by fluorescence-activated cell sorting (FACS) for the expression of GluN1. Our results show that associative learning (tone with shock) induces an increase in GluN1 at vHPC-to-IL synaptosomes in males. In contrast, CFC did not affect GluN1 expression. In addition, neither CFC nor AFC induced changes in GluN1 expression in the females, suggesting that this synaptic plasticity is sex-dependent.

## Materials and Methods

### Labeling Ventral Hippocampal Synapses With EYFP

Male and female Sprague–Dawley rats (P30) received infusions of an AAV5 vector (1.0 μL per hemisphere) that promotes channelrhodopsin-2 (ChR) and enhanced yellow fluorescent protein expression driven by the glutamatergic neuron-specific CaMKIIα promoter [AAV-CaMKIIα-hChR2(H134R)-EYFP; University of North Carolina at Chapel Hill Vector Core Services]. A titer of 10^11^ particles was infused into the vHPC with a 5-μL Hamilton syringe using the following stereotactic coordinates (−5.50 mm AP; ±4.50 mm ML; −7.0 mm DV). After a recovery of at least 2 months for optimal expression of ChR-EYFP, the rats were arbitrarily assigned to different experimental groups. Every animal used in experiments 1 and 2 expressed ChR-EYFP for tracing vHPC-to-IL projections, since this protein is trafficked to the axon terminals. We did not use the ChR-EYFP for optogenetic stimulation. After sacrifice, we took images with either an epifluorescence Olympus BX60 microscope or a NikonC2+ confocal microscope of random samples to confirm that EYFP expression was restricted to the vHPC and EYFP-labeled axons were visible in IL (Supplementary Figure 5).

### Behavioral Groups for Experiment 1

Adult male and female rats (P90) were divided into three groups and placed in operant chambers (Ugo Basile) with three frosted white sides, a clear front, and a metal grid floor to provide electric foot shocks. The exposure group (EXPO) was exposed to the chambers for the same length of time without receiving any electric foot shocks. Animals were not habituated to the conditioning chambers. The CFC group was allowed to explore the chamber for 2 min to establish baseline movement of each animal in that context. Then, this group received five electric foot shocks (0.7 mA) spaced 2 min apart. The AFC group received six tones (Hz, 80 dB, 30 s) with an interval of 120 s between tones. Tones 2–6 were paired with a mild electrical foot shock (conditioned stimulus, CS, 0.44 mA, 0.5 s) beginning at the end of each tone. The next day, all rats were placed back into the same training context to test their recall memory. The AFC group received two tones, and the EXPO and CFC groups only received context exposure.

### Behavioral Groups for Experiment 2

Adult male (P90, P180, and P330) and female (P90) rats were divided into three groups and placed in operant chambers (Ugo Basile). The older ages in the male group were caused by unexpected delays. Animals were not habituated to the conditioning chambers. On day 1, the AFC and extinction (EXT) groups received six tones (Hz, 80 dB, 30 s) with an interval of 120 s between tones. Tones 2–6 were paired with an electrical foot shock (conditioned stimulus, CS, 0.44 mA, 0.5 s) beginning at the end of each tone. On day 1, the control pseudoconditioned (PSUEDO) group received the six auditory tones unpaired with electric foot shocks and then five rapid electric foot shocks immediately before removing them from the conditioning chamber to control for tone and shock exposure without inducing conditioned inhibition. On day 2, the PSEUDO and AFC groups remained in their home cages and the EXT group received two sessions of 15 tones (30 s duration, 2 min interval between tones) separated by 1 h. The third day, all groups were placed back into the same training context and were given two tones to test their recall memory.

### Synaptosome Isolation

All behaviors and sacrifices were done during the morning. Immediately after recall, the animals received an overdose of 1.0 mL of pentobarbital mixed with phenytoin sodium (Euthanasia-III, MED-PHARMEX Inc., Pomona, CA, United States). After brain extraction, the brains were coronal cut into 1 mm slices with a brain matrix (BS-A 6000C, BrainTree Scientific, Inc.). IL tissue punches (1.5 mm) from IL were taken from each rat. Tissue punches were homogenized, and protein extraction was performed to extract synaptic proteins using the Syn-PER Synaptic Protein Extraction Reagent (cat no. 87793, Thermo Scientific) with phosphatase and protease inhibitors to avoid the degradation of proteins (P5726-5ML, Sigma and P2714-1BTL, Sigma). After homogenization, the samples were centrifuged for 10 min at 3,600 rpm at 4°C. The supernatant was centrifuged a second time at 11,400 rpm for 20 min at 4°C and the supernatant was discarded, and the pellet was kept. Meanwhile, antibodies against an extracellular epitope of rat GluN1 (cat no. AGC-001, Alomone Lab Co.) were conjugated with Alexa Fluor 647 following the manufacturer’s instructions (Antibody Labeling Kit, cat no. A20186, Ex/Em 650/668). Before using the antibodies on the experimental samples, each batch of labeled antibody was tested to ensure the detection of GluN1 on synaptosomes from a naïve rat with appropriate fluorescence. The antibody was diluted (1:100) in 5% bovine serum albumin (BSA) in PBS and incubated with the synaptosomes for 1 h on ice. Then, the synaptosomes were washed once with 600 uL of 5% BSA in PBS and centrifuged for 5 min at 5,400 rpm at 4°C to wash away unbound antibody. The supernatant was discarded and the pellet was suspended in 400 uL of 0.5% paraformaldehyde, protected from light, and stored at 4°C.

### FACS

Once the vHPC-to-IL synapses were labeled with AAV-mediated expression of EYFP and synaptosomes were isolated from IL tissue punches, EYFP-expressing synaptosomes were identified *via* FACS. First, we identified the appropriate size gate for our FACS machine (BD FACSAria I; BD Biosciences, San Jose, CA, United States) with fluorescent beads of 1, 2, and 4 μm diameters. Synaptosomes fall within this size range (Gylys et al., 2004; Evans, 2015) and larger particles are more likely to have intact presynaptic and postsynaptic membranes (Gylys et al., 2004). Then, synaptosomes from IL tissue punches were selected using these size limits and vHPC-to-IL synaptosomes were identified by EYFP fluorescence. Next, the expression of GluN1 by the EYFP+ population of synaptosomes was determined by detecting Alexa Fluor 647 which was conjugated to the anti-GluN1 antibody (Alexa Fluor 645 Antibody Labeling Kit, cat no. A20186). The gate for EYFP that represents the channelrhodopsin from the virus was established by comparing the detection of fluorescence in the 488 nm wavelength in synaptosomes from an animal that was not injected with the viral vector with synaptosomes from an AAV-injected animal. Similarly, synaptosomes from a rat that did not express EYFP were incubated with the conjugated anti-GluN1 antibody and the detectible fluorescence at 645 nm was compared to unlabeled synaptosomes to set the gate for detection of GluN1+ synaptosomes. To measure the mean fluorescence intensity of GluN1 immunolabeling for each rat, we generated a histogram of the double-positive EYFP+/GluN1+ synaptosomes in FlowJo software. Markers were placed to at the beginning and the end of the histogram, and the mean, median, and mode of the GluN1 fluorescence intensity were automatically calculated by FlowJo. The forward scatter of the double-positive EYFP+/GluN1+ synaptosomes was measured in FlowJo as an estimate of relative difference in the size of the synaptosomes. The side scatter of the double-positive EYFP+/GluN1+ synaptosomes was measured in FlowJo as an estimate of relative difference in the internal complexity (i.e., granularity and internal structures) of the synaptosomes.

Recent studies found that it is important to consider the potential contribution of aggregates of two or more synaptosomes in flow cytometry experiments (Biesemann et al., 2014; Hobson and Sims, 2019). These articles demonstrate that under proper conditions, the contribution of aggregates can be reduced to insignificant levels. To determine the contribution of aggregates in our samples, we isolated IL synaptosomes, separated them into two tubes, and incubated each sample with a different antibody conjugated to a different fluorophore. After immunolabeling, the samples were fixed, mixed together, and analyzed by FACS. As shown in Supplementary Figure 1, the FACS analysis found negligible amounts of double-positive synaptosomes which would represent aggregates of individually labeled synaptosomes. These data suggest that our experimental process generates minimal aggregates which do not contribute significatively to our results.

### Statistical Analysis

In experiment 1, contextual fear was measured in the CFC group as the percent of time spent freezing during the 60 s before the shock in the CFC group and during the equivalent period in the EXPO group during training on day 1 with ANY-maze software (Ugo Basile, Italy). During recall, the freezing during 2 min in the conditioning context was measured as contextual fear in the CFC and EXPO groups. In the PSEUDO group, contextual fear was measured as freezing during 30 s before the first tone in the conditioning chamber during recall. In the PSEUDO, AFC, and EXT groups, auditory fear was measured as the percent of time spent freezing during each 30 s tone of training and recall with ANY-maze software. Behavioral data acquired on day 1 in experiments 1 and 2 were analyzed by repeated-measures two-way analysis of variance (ANOVA) followed by Tukey *post hoc* test (Graphpad Prism version 9.1.0, San Diego, CA, United States). The two trials of fear recall were averaged and compared by one-way ANOVA followed by Tukey *post hoc* test by treatment and sex (Graphpad Prism). Significance was set at *p* ≤ 0.05. The raw data obtained from the BD FACSAria I (BD Biosciences, San Jose, CA, United States) were filtered by size and EYFP expression using FlowJo 2 (BD Life Sciences, Becton, Dickinson & Company). The resulting EYFP+ population of synaptosomes was subsequently analyzed for immunolabeling of GluN1 in FlowJo. The fluorescence intensity histograms were generated by selecting the EYFP+/GluN1+ synaptosomes in quadrant 2 and analyzing them in FlowJo. A similar number of EYFP+/GluN1+ IL synaptosomes were detected and analyzed in both sexes [males: 7170 ± SEM: females: 8814 ± SEM; U (648, 528) = 203, *p* = 0.08]. The data were categorized as non-parametric by Shapiro–Wilk. Statistical analysis was performed with Kruskal–Wallis non-parametric test followed by Dunn’s multiple comparison test (Graphpad Prism version 9.1.0, San Diego, CA, United States). Significance was set at *p* ≤ 0.05. The investigators were not completely blinded to the treatment group. However, most of the samples were identified by a rat ID# and run by a FACS technician who was blinded to the treatment. The resulting data were then analyzed in FlowJo and saved as a PDF before the results were separated into treatment groups.

## Results

### Fear-Related Behaviors for Experiment 1

**FIGURE 1:**
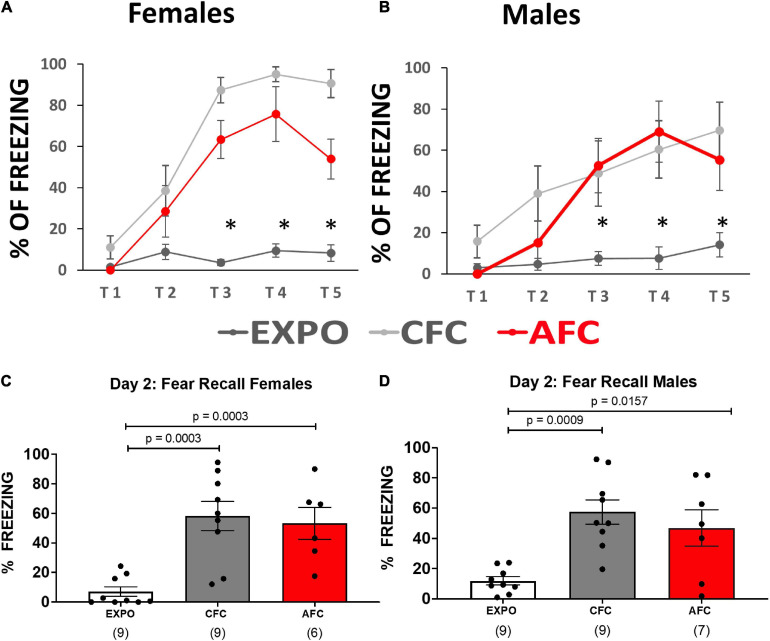
Female and male rats acquired contextual and auditory fear. **(A,B)** Fear responses during training in the exposure, CFC, and AFC females and males, respectively. Asterisk denotes trials in which the CFC and AFC groups show more freezing than the exposure group (*p* ≤ 0.05). **(C,D)** Long-term memory on day 2 of female and male groups showing freezing to the context in the EXPO and CFC groups and to the tone in the AFC group. Sample sizes are indicated below each bar in parentheses.

Male and female rats were injected with an AAV vector to induce expression of channelrhodopsin and EYFP in vHPC neurons. After waiting 2 months for optimal expression and transport of the EYFP to the axon terminals of the vHPC neurons, rats (P90) were arbitrarily divided into three groups. The three experimental groups were designed to test whether contextual or AFC changes NMDA receptor expression at vHPC synapses in IL (Figure 1). The CFC group (*n* = 9 females and 9 males) received five foot shocks in the conditioning chamber, the exposure group (EXPO, *n* = 9 females and 9 males) was placed in the conditioning chamber and did not receive foot shocks, and the AFC group (*n* = 6 females and 8 males) received one habituation tone and five tones paired with a foot shock (Figures 1A,B, respectively). The female and male rats in the CFC and AFC groups showed a gradual increase in freezing during the behavioral protocol, indicating that the groups acquired contextual and auditory fear, respectively (Figures 1A,B and Supplementary Statistical Table). In contrast, the EXPO group maintained a low level of freezing throughout.

The following day the long-term memory was analyzed by measuring freezing during the first minute in the conditioning context in the EXPO and CFC groups or during two tones in the AFC group. Both females and males showed similar patterns of learning fear context per group (Figures 1C,D). Both the male [*F*(2,22) = 3.954, *p* = 0.0341] and female groups [*F*(2,21) = 3.608, *p* = 0.0450] showed different levels of freezing to the tones. The CFC (male, *p* = 0.0009; female, *p* = 0.0003) and AFC (male, *p* = 0.0157; female, *p* = 0.0029) groups showed more freezing than the EXPO groups, indicating that male and female rats recalled their fear learning from the previous day.

### AFC Increases GluN1 Expression at vHPC-to-IL Synaptosomes

**FIGURE 2:**
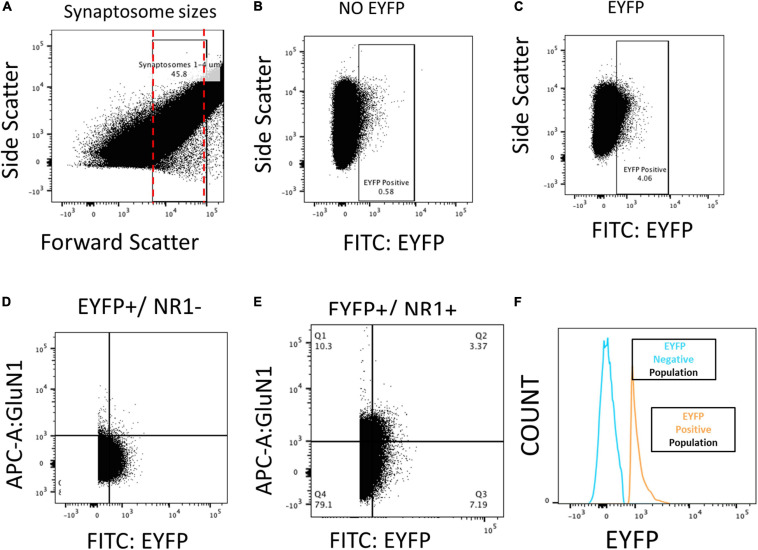
FACS analysis of the NMDA subunit GluN1 on vHPC-to-IL synaptosomes from male and female rats. **(A)** Particles between 1 and 4 μm were selected as synaptosomes after isolation from IL tissue and used for subsequent analysis. **(B)** Rats without virus (NO EYFP) had a low percentage of synaptosomes in the EYFP+ quadrant. **(C)** Rats injected with the EYFP-expressing virus into the VHPC showed abundant expression of EYFP+ synaptosomes isolated from IL tissue. **(D)** EYFP+/GluN1– samples that lacked the antibody against GluN1 show no expression in GluN1+ quadrants. **(E,F)** EYFP+/GluN1+ samples show abundant detection of EYFP+/GluN1+ synaptosomes, indicating that we could isolate and quantify GluN1 expression in VHPC-to-IL synaptosomes.

After the fear recall on day 2, the animals were sacrificed, brain slices were cut, and tissue punches were extracted from IL. The samples were homogenized, and synaptosomes were isolated by centrifugation and incubated with an antibody against an extracellular epitope of GluN1 (cat no. AGC-001, Alomone Lab Co.). Labeled synaptosomes were then analyzed by FACS. Figure 2 shows the filters applied to the FACS data to measure GluN1 expression on vHPC-to-IL synaptosomes. First, the results were filtered by size to select the particles within the 1–4 μm size range of synaptosomes (Figure 2A). Then vHPC synaptosomes were identified by EYFP expression (Figure 2C) which was not found in synaptosomes from rats that did not receive viral injections (Figure 2B) confirming the capacity to detect the EYFP expressed in vHPC axon terminals. Finally, the double-positive synaptosomes (EYFP+/GluN1+) were identified to measure GluN1 expression (Figures 2D,E). A histogram shows that synaptosomes expressing EYFP could be separated from synaptosomes not expressing EYFP (Figure 2F).

**FIGURE 3:**
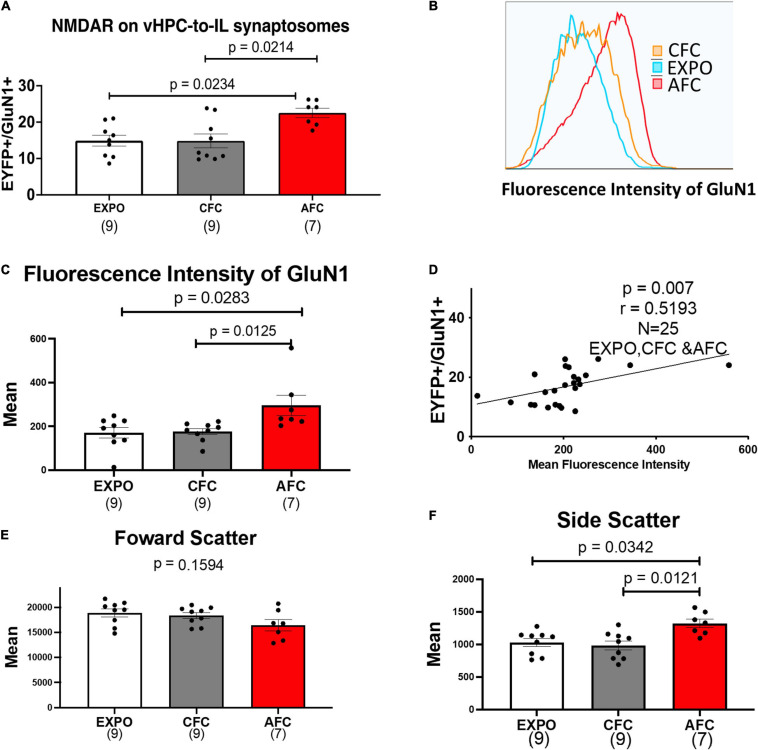
AFC increases the number of NMDARS on VHPC-to-IL synaptosomes in males. **(A)** Relative levels of EYFP+/GluN1+ synaptosomes in male behavioral groups. **(B)** Representative histogram of the fluorescence intensity of GluN1 on EYFP+/GluN1+ synaptosomes from a single sample from the Exposure, CFC, and AFC male groups. **(C)** Mean fluorescence intensity of GluN1 on EYFP+/GluN1+ synaptosomes in male groups. **(D)** Correlation between the levels of EYFP+/GluN1+ synaptosomes and the mean fluorescence intensity of GluN1 in the male samples. **(E)** Mean forward scatter of the EYFP+/GluN1+ synaptosomes in the male groups. **(F)** Mean side scatter of the EYFP+/GluN1+ synaptosomes in the male groups. Sample sizes are indicated below each bar.

Next, we analyzed the expression of EYFP+/GluN1+ synaptosomes in IL tissue from the different behavioral groups. Figure 3A shows that fear acquisition affected the expression of GluN1 on EYFP+ vHPC-to-IL synaptosomes in males. The expression of GluN1 on EYFP+ vHPC-to-IL synaptosomes varied in the male groups [*H*(2,22) = 9.166, *p* = 0.0102; Figure 3A]. The male AFC group showed more EYFP+/GluN1+ synaptosomes than in the EXPO (*p* = 0.0234) or CFC groups (*p* = 0.0214) suggesting that AFC increases NMDA receptor expression at vHPC-to-IL synapses. The CFC group expressed similar levels to the EXPO group (*p* > 0.9999), suggesting that the contextual fear acquisition was insufficient to induce the change.

To explore whether the increase in the number of EYFP+/GluN1+ synaptosomes was caused by synaptogenesis or an increase in NMDA receptors in existing vHPC-to-IL synapses, we compared histograms of the fluorescence intensity of GluN1 labeled EYFP+ synaptosomes in the EXPO, CFC, and AFC groups of male rats (Figures 3B,C). We found that AFC histograms were shifted to the right compared to the EXPO and CFC histograms producing a difference in the mean fluorescent intensity of GluN1 on EYFP+ vHPC-to-IL synaptosomes in the male groups [*H*(2,22) = 9.625, *p* = 0.0081]. The mean of the AFC group was greater than the EXPO (*p* = 0.0283) and the CFC group (*p* = 0.0125). These data suggest that AFC increased the number of NMDARs at vHPC-to-IL synapses rather than simply increasing the number of vHPC-to-IL synapses. Furthermore, we found a correlation between the number of EYFP+/GluN1+ synaptosomes and the mean GluN1 fluorescence intensity (*r* = 0.5193, *p* = 0.0078; Figure 3D).

The observed increase in NMDA receptors on synaptosomes could occur if AFC increased the size of the synapses. To test this possibility, we analyzed the forward scatter of the EYFP+/GluN1+ synaptosomes since larger synaptosomes would produce more forward scatter. We found that the forward scatter was similar in all groups, suggesting that the synaptosomes are similar in size and that fear learning did not increase the size of the vHPC-to-IL synapses [*H*(2,22) = 3.673, *p* = 0.1594; Figure 3E]. However, the side scatter did vary among groups [*H*(2,22) = 9.485, *p* = 0.0087; Figure 3F]. We observed an increase in the side scatter of the AFC group compared to the EXPO (*p* = 0.0342) and CFC groups (*p* = 0.0121), suggesting that fear learning increased the intracellular complexity of the vHPC-IL synaptosomes.

**FIGURE 4:**
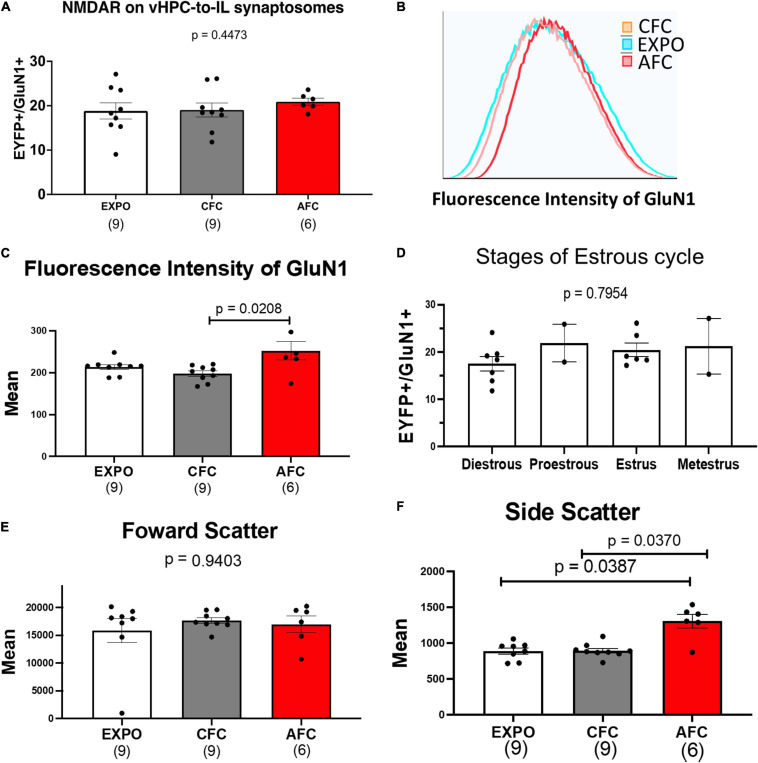
AFC does not alter GluN1 expression at vHPC-IL synaptosomes in females. **(A)** Relative levels of EYFP+/GluN1+ synaptosomes in female behavioral groups. **(B)** Representative histogram of the fluorescence intensity of GluN1 on EYFP+/GluN1+ synaptosomes from a single sample from the EXPO, CFC, and AFC female groups. **(C)** Mean fluorescence intensity of GluN1 on EYFP+/GluN1+ synaptosomes in female groups. **(D)** Mean fluorescence intensity of GluN1 on EYFP+/GluN1+ synaptosomes across the estrous cycle. **(E)** Mean forward scatter of the EYFP+/GluN1+ synaptosomes in the female groups. **(F)** Mean side scatter of the EYFP+/GluN1+ synaptosomes in the female groups. Sample sizes are indicated below each bar.

In contrast to the male groups, we found similar GluN1 expression in all female behavioral groups suggesting that fear acquisition does not alter NMDA receptors at vHPC-to-IL synapses in females [*H*(2,22) = 1.609, *p* = 0.44; Figure 4A]. The histograms of the fluorescence intensity of GluN1 labeled EYFP+ synaptosomes were also similar in the EXPO, CFC, and AFC groups of female rats (Figure 4B). Although we found that the mean fluorescent intensity of GluN1 on EYFP+ vHPC-to-IL synaptosomes was different in the female groups [*H*(2,22) = 7.951, *p* = 0.0188], the only difference was between the AFC and CFC groups (*p* = 0.0161; Figure 4C). The mean of the EXPO group was similar to the CFC (*p* = 0.3190) and the AFC group (*p* = 0.6195), suggesting that neither contextual nor auditory fear acquisition altered GluN1 expression on vHPC-to-IL synaptosomes in the females. Furthermore, we found no correlation between the number of EYFP+/GluN1+ synaptosomes and the mean GluN1 fluorescence intensity (*r* = 0.08, *p* = 0.1065; Supplementary Figure 2). We also examined whether the GluN1 expression in females varied with the estrous cycle that was determined by vaginal smears at the time of sacrifice on day 2. We found no difference in mean fluorescent intensity of GluN1 labeled EYFP+ synaptosomes across the estrous cycle of the rats [*H*(2,22) = 0.5232, *p* = 0.7869; Figure 4D]. The number of EYFP+/GluN1+ synaptosomes did not also vary across the estrous cycle [*H*(2,22) = 9.624, *p* = 0.2; Supplementary Figure 2C]. This suggests that cyclic changes in estrogen and other reproductive hormones did not alter the NMDA receptor expression on the vHPC-to-IL synapses.

The forward scatter of the EYFP+/GluN1+ synaptosomes was similar in all female groups, suggesting that the synaptosomes are similar in size and that fear learning did not change the size of the vHPC-to-IL synapses in females [*H*(2,22) = 0.1231, *p* = 0.9403; Figure 4E]. As in males, we observed an increase in the side scatter of the AFC group compared to the CFC group (*p* = 0.0370) and the EXPO group (*p* = 0.0387), suggesting that auditory fear learning changed the intracellular complexity of the vHPC-IL synaptosomes [*H*(2,22) = 6.605, *p* = 0.0368; Figure 4F]. To explore the possibility that the females and males had different basal levels of GluN1 expression at this synapse, we compared the GluN1 expression in the EXPO group of both sexes. The female and male EXPO groups expressed similar levels of GluN1, suggesting that both sexes express similar basal levels of GluN1 (Mann–Whitney U = 23, *p* = 0.14).

### Fear Behavior for Experiment II

**FIGURE 5:**
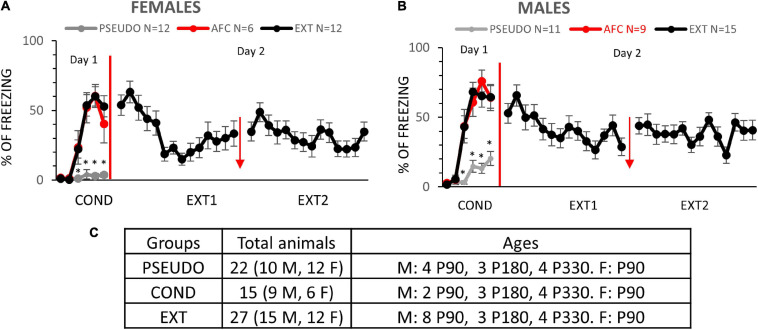
Females and males learn AFC and fear extinction. **(A,B)** Fear responses during training in the PSEUDO, AFC, and EXT female and male groups, respectively. Red arrow indicates 1 h break between extinction sessions. **(C)** Distribution of animals by age and sex in experiment 2. * Denotes trials in which the AFC and EXT groups show more freezing than the PSEUDO group (*p* ≤ 0.05).

Our initial experiment suggests that AFC increases the expression of NMDARs at vHPC-to-IL synapses of male rats. To determine if this synaptic plasticity required associative learning and whether fear extinction could reverse the synaptic changes, we repeated our experiments with a different cohort of rats. We divided male and female rats into three different behavioral groups, pseudoconditioning (PSEUDO), AFC, and extinction (EXT) (Figure 5C). Due to unexpected delays, the male rats were different ages (P90, P180, and P330) at the beginning of the behavioral training. As in the previous experiment, rats received injections of AAV into the vHPC to express ChR-EYFP at least 2 months before behavioral training. On day 1, the PSEUDO groups (*n* = 11 males and 12 females) received six tones and then five quick foot shocks immediately before being removed from the conditioning chamber to control for tone and shock exposure without inducing conditioned inhibition (Figures 5A,B). On day 1, the AFC groups (*n* = 9 males and 6 females) and the EXT groups (*n* = 15 males and 12 females) received one habituation tone and five tones paired with a foot shock. Both female and male rats in the AFC and EXT groups showed a gradual increase in freezing on day 1, indicating that they acquired fear to the tone (Supplementary Statistical Table). In contrast, the PSEUDO group that did not receive paired tones and shocks presented a low level of freezing to the tone (Figures 5A,B). On day 2, the EXT groups received two sessions of 14 tones without foot shocks in the conditioning context with 1 h between sessions.

**FIGURE 6:**
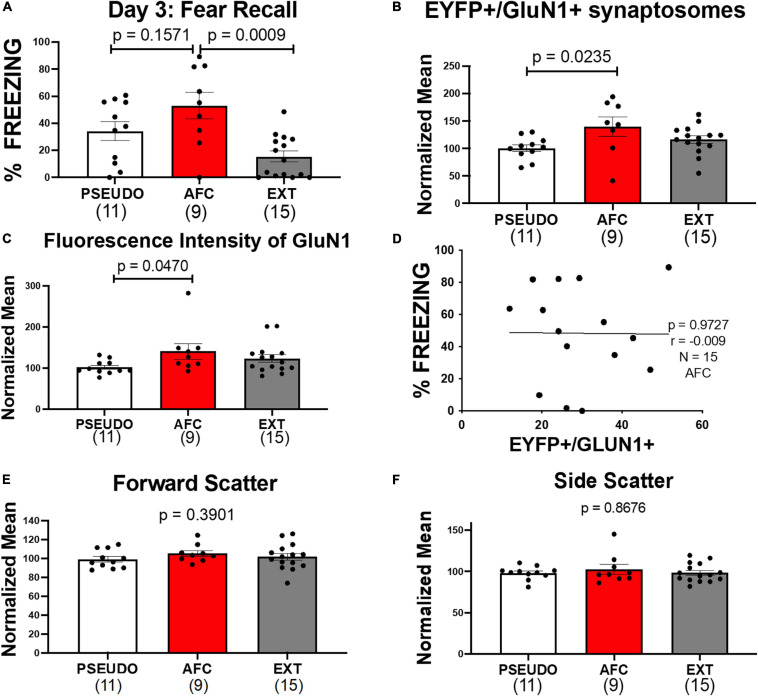
Associative fear learning increases NMDARS on VHPC-to-IL synaptosomes in male rats. **(A)** Fear recall in the male groups. **(B)** Levels of EYFP+/GluN1+ synaptosomes normalized to the mean of the PSEUDO group of the corresponding age in the male groups. **(C)** Mean fluorescence intensity of GluN1 on EYFP+/GluN1+ synaptosomes in male groups normalized to the mean of the PSEUDO group of the corresponding age. **(D)** Graph comparing the levels of EYFP+/GluN1+ synaptosomes and the % freezing during recall of the males in the AFC groups in experiments 1 and 2. **(E,F)** Forward scatter and side scatter of EYFP+/GluN1+ synaptosomes in male groups normalized to the mean of the PSEUDO group of the corresponding age.

On day 3, we tested the long-term memory of all three groups by exposing the rats to two tones in the conditioning context. In the males, the groups showed different levels of freezing to the tones [*F*(2,32) = 2.245, *p* = 0.0012; Figure 6A]. The AFC group only showed a trend of higher freezing than the PSEUDO group, since the unpaired PSEUDO group showed freezing during the tone (*p* = 0.1571). To determine if the PSEUDO group was freezing to the tone or to the context, we compared freezing during the 30 s before the first tone (contextual fear) to the freezing during each of the two recall tones (Supplementary Figure 3A). The rats froze similar amounts during the pretone and tone exposure periods [*F*(2,27) = 0.7582, *p* = 0.4782], suggesting that the PSEUDO males were actually showing contextual fear during the tone. The EXT group showed less freezing than the AFC group indicating good recall of fear extinction (*p* = 0.0009).

Next we analyzed the expression of GluN1 on vHPC-to-IL synaptosomes in the male groups and found a trend of a difference in the groups [*H*(2,32) = 4.526, *p* = 0.1040; Supplementary Figure 3B]. Since we used male rats from P90, P180, and P330 in this experiment (Figure 5C), any learning-induced changes could be obscured by changes due to aging or time of ChR-EYFP expression. Therefore, we tested whether the expression of GluN1 at vHPC-to-IL synaptosomes varied with age in the male PSEUDO group which serves as our baseline group. As shown in Supplementary Figure 3C, the number of EYFP+/GluN1+ synaptosomes trended to be higher [*H*(2,9) = 5.182, *p* = 0.0684] and the fluorescent intensity of GluN1 immunolabeling increased in older male rats [*H*(2,9) = 7.03, *p* = 0.0142]. The side scatter of the EYFP+/GluN1+ synaptosomes also increased with age [*H*(2,9) = 7.477, *p* = 0.0062], but the forward scatter remained constant across ages [*H*(2,9) = 1.417, *p* = 0.5238]. To adjust for the effect of age, we normalized the EYFP+/GluN1+ expression and GluN1 fluorescent intensity to the average of the PSEUDO group of the same age (Figures 6B,C). We separated the male groups by age and then normalized all data from P90 male animals to the average of the P90 PSEUDO group, all data from the P180 animals to the average of the P180 PSEUDO group, and all the data from P330 animals to the average of the P330 PSEUDO group. Then we combined the normalized data into the PSEUDO, AFC, and Ext groups. We found that the normalized EYFP+/GluN1+ expression [*H*(2,32) = 7.182, *p* = 0.0276] and GluN1 fluorescent intensity [*H*(2,32) = 6.201, *p* = 0.0450] varied among groups. To test whether associative learning was required for the changes in GluN1 expression, we compared the male PSEUDO and AFC groups. The results show that the AFC group expressed more EYFP+/GluN1+ synaptosomes than the PSEUDO group (*p* = 0.0235; Figure 6B). The AFC group also showed greater fluorescent intensity of GluN1 immunolabeling than the PSEUDO group (*p* = 0.0470; Figure 6C). These data suggest that synapses from vHPC-to-IL had more NMDARs after AFC. Therefore, the increase in GluN1 expression at vHPC-to-IL synapses observed in experiment 1 was replicated in this new group of rats, suggesting that the findings are robust. Even though the PSEUDO group showed similar levels of freezing during the tones as the AFC group, the PSEUDO group showed fewer EYFP+/GluN1+ synaptosomes than the AFC group. This suggests that the GluN1 expression was altered by the tone-shock pairing rather than the increase in fear *per se*.

The increased expression of GluN1 could contribute to the encoding of the AFC memory. To further examine this possibility, we compared GluN1 expression in the male AFC groups from experiments 1 and 2 to their fear level during recall. We found that the freezing at recall did not correlate with GluN1 expression, suggesting that the increase in NMDA receptors may not encode fear memory *per se* (*r* = −0.0097, *p* = 0.9727, *N* = 15; Figure 6D). Furthermore, the freezing to the last tone of conditioning on day 1 did not also correlate with GluN1 expression (*r* = 0.1, *p* = 0.7, *N* = 15).

Next, we compared the AFC and EXT groups to determine if extinction reversed the increase of NMDAR. The molecular assessment shows that the AFC and EXT groups expressed similar levels of EYFP+/GluN1+ synaptosomes (*p* = 0.5080; Figure 6B) and GluN1 immunofluorescence (*p* > 0.9999; Figure 6C). This suggests that EXT reduced the conditioned fear behavior without reversing the increase in GluN1 at vHPC-to-IL synapses induced by AFC in males. In addition, we analyzed forward and side scatter of the EYFP+/GluN1+ synaptosomes. PSEUDO, AFC, and EXT groups had similar values of forward [*H*(2,32) = 1.882, *p* = 0.3901] and side scatter [*H*(2,32) = 0.2840, *p* = 0.8676], suggesting that the synaptosomes were similar in size and complexity (Figures 6E,F).

**FIGURE 7:**
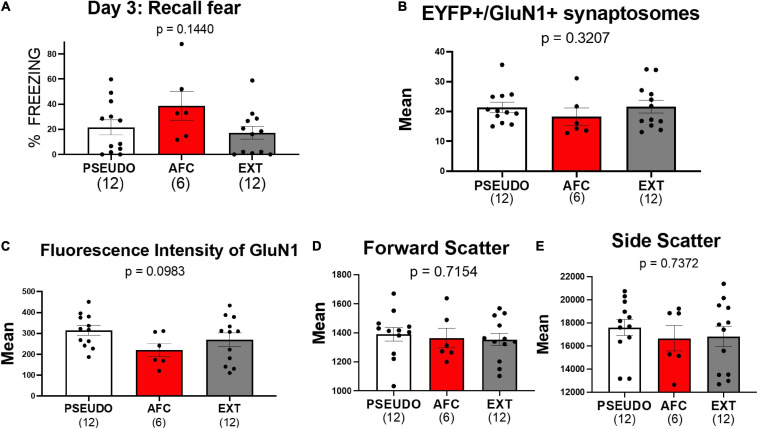
Associative fear learning does not increase NMDAR on vHPC-to-IL synaptosomes in female rats. **(A)** Fear recall in the female groups. **(B)** Levels of EYFP+/GluN1+ synaptosomes in the female groups. **(C–E)** Mean fluorescence intensity of GluN1, forward scatter, and side scatter of EYFP+/GluN1+ synaptosomes in female groups.

Although we did not see any changes in GluN1 expression in the females in experiment 1, we also compared the GluN1 expression on vHPC-to-IL synaptosomes in female PSEUDO, AFC, and EXT groups (Figure 7). In the females, the groups showed similar levels of freezing to the tones [*F*(2,27) = 0.4872, *p* = 0.1440; Figure 7A). As with the males, the PSEUDO group showed abundant freezing during the recall tones which obscured any effect of AFC. To determine if the female PSEUDO group was freezing to the tone or to the context, we compared freezing during the 30 s before the first tone (contextual fear) to the freezing during each of the two recall tones (Supplementary Figure 4A). The female rats froze less during the pretone than during the second (*p* = 0.0112) tone exposure period, suggesting that the PSEUDO females were actually showing fear to the tone. Therefore, the PSEUDO protocol appears to have induced AFC in some of the female rats. In addition, the AFC and EXT groups showed similar levels of freezing suggesting poor extinction recall in the females.

Consistent with the results of experiment 1, we did not find any differences in EYFP+/GluN1+ expression [*H*(2,27) = 2.274, *p* = 0.3207; Figure 7B], GluN1 fluorescent intensity [*H*(2,27) = 4.639, *p* = 0.0983; Figure 7C], forward scatter [*H*(2,27) = 0.6699, *p* = 0.7154; Figure 7D], or side scatter [*H*(2,27) = 0.6097, *p* = 0.7372; Figure 7E]. However, the lack of behavioral differences among the female groups may have obscured any difference in GluN1 expression. Therefore, we also tested whether removing three animals from the PSEUDO group and one animal from the EXT group that exhibited freezing above 40% to create a behavior difference among the groups would similarly create a difference in GluN1 expression. As shown in Supplementary Figure 4, removing these animals created a behavioral difference among the groups but did not show any difference in GluN1 expression among the female groups. These data further suggest that fear learning does not alter NMDAR at the vHPC-to-IL synapse in females.

## Discussion

**FIGURE 8:**
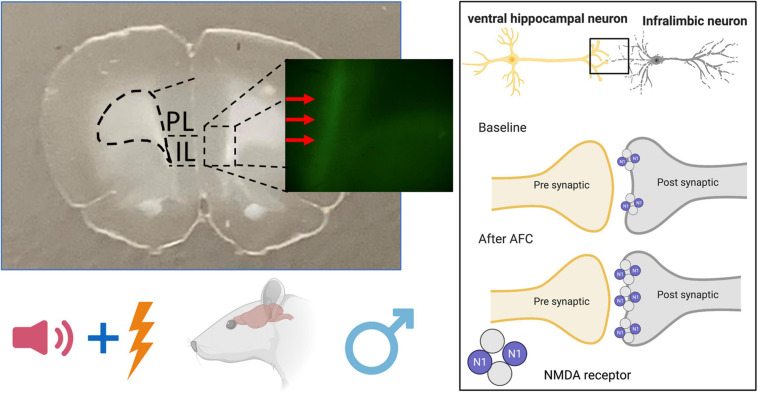
Schematic diagram of NMDAR changes after auditory fear conditioning in male rat brain. Associative fear learning increases NMDAR on ventral hippocampal projections to the infralimbic cortex.

Scientists frequently employ the AAV expression of ChR-EYFP to determine the role of specific neuronal projections in behavior. In this study, we used FACS analysis of ChR-EYFP-labeled vHPC-to-IL synaptosomes to detect changes in GluN1 expression induced by fear conditioning. Our main finding is that AFC increased GluN1 expression at this synapse in males compared to EXPO and CFC, suggesting that the tone-shock pairing induced the change (Figure 8). In further support, male rats in the PSEUDO group that received unpaired tones and shocks did not exhibit the increase in GluN1 at vHPC-to-IL synaptosomes. In addition, the increase in GluN1 expression was not reversed by extinction. In contrast to the males, the females did not show differences in GluN1 expression at this synapse after any fear paradigm, suggesting that this is a sex-dependent form of synaptic plasticity induced by AFC in IL.

Our findings extend previous research showing that AFC induces synapse-dependent synaptic plasticity in IL of male rodents (Pattwell et al., 2012; Sepulveda-Orengo et al., 2013; Soler-Cedeño et al., 2019). In contrast to the electrophysiological finding of less NMDAR current after AFC reported earlier (Soler-Cedeño et al., 2019), we observed an increase in the fraction of vHPC-to-IL synaptosomes that expressed detectable levels of the obligatory GluN1 subunit of NMDAR after AFC. This increase in NMDAR at vHPC-to-IL synapses required the animal to learn the association of the cue and the shock, since neither CFC nor unpaired tones and shocks were sufficient to produce the change. The mean fluorescence intensity of GluN1 on the vHPC-to-IL synaptosomes also increased after AFC, suggesting that the associative learning led to more NMDARs at these synapses. The increase in the number of NMDARs occurred without an increase in synapse size, since the forward scatter of the synaptosomes did not change. However, the side scatter of the synaptosomes also increased after AFC suggesting a change in the intracellular organelles of the synaptosomes (Biesemann et al., 2014).

The vast majority of previous studies have used only male rodents to examine molecular mechanisms underlying fear learning (Zinebi et al., 2003; Burgos-Robles et al., 2007; Pattwell et al., 2012; Paoletti et al., 2013; Sepulveda-Orengo et al., 2013; Soler-Cedeño et al., 2019). Therefore, we also examined whether fear acquisition induces similar plasticity in females. In contrast to the plasticity observed in males, the females did not show any differences in the fraction of EYFP+/GluN1+ synaptosomes, GluN1 fluorescence intensity, or side scatter. Since we included females at different stages of the estrous cycle, it is possible that variability in the measurements could obscure any differences. However, the lack of strong correlation with the estrous stage argues against this possibility. The females did learn similar levels of conditioned fear to the tones. These data imply sex differences in the molecular mechanisms that encode auditory fear learning that warrants further study. Furthermore, this raises the possibility that many of the previous identified molecular mechanisms of fear acquisition and extinction may not apply to females.

The increase in NMDAR at vHPC-to-IL synaptosomes after associative fear learning does not seem to encode fear *per se*. First, the levels of GluN1 did not correlate with fear recall or fear acquisition. Second, the changes in GluN1 were not reversed by fear extinction, although fear extinction did reduce fear expression. Third, in spite of receiving unpaired tones and shocks, the PSEUDO group showed fear to the tone during recall that was similar to the fear expressed by the AFC group but only the AFC group showed the increase in GluN1 on the vHPC-to-IL synaptosomes. Taken together, these findings suggest that although learning the association between tone and shock is required to create these molecular changes, the changes do not encode the conditioned fear.

More NMDAR in vHPC-to-IL after AFC could produce a number of physiological consequences. The increase of NMDAR could enhance calcium influx into the dendritic spines to facilitate subsequence learning or memory formation. For example, animals might acquire more robust extinction memories due to more calcium influx through NMDAR during extinction learning. Consistent with this, increased NMDARs in IL synapses are associated with enhanced synaptic plasticity and increased fear extinction memory (Abumaria et al., 2011). In addition, animals with good fear extinction recall show more NMDAR-dependent bursts of action potentials in IL the day of extinction learning and blocking NMDAR in IL during extinction learning impairs the long-term extinction memory (Burgos-Robles et al., 2007). Furthermore, researchers found that stimulating NMDARs with D-cycloserine, a partial agonist at the glycine binding site of NMDARs, can enhance fear extinction in rodents (Richardson et al., 2004) and humans (Morrison and Ressler, 2014). Therefore, the literature highlights the importance of NMDARs for fear extinction and supports the possibility that the increase in NMDARs on the vHPC-to-IL synapses could contribute to fear extinction in the males.

Whole-cell recordings of optically stimulated vHPC synapses in IL found less synaptic NMDA current in layer V pyramidal neurons after AFC in males (Soler-Cedeño et al., 2019). Therefore, we expected to observe a reduction in the NMDARs on the vHPC-to-IL synaptosomes. In contrast, our results show increased NMDAR. Several possibilities could reconcile these apparently conflicting results. One possibility is that AFC might cause less vHPC activation of NMDA currents in IL neurons through dynamic changes in NMDAR subunit composition or phosphorylation state instead of a reduction in the relative amount of NMDAR (Lopez de and Sah, 2003; Zinebi et al., 2003; Abumaria et al., 2011; Lussier et al., 2015). The electrophysiological study measured the NMDAR current during the decay phase rather than at the peak to reduce AMPAR current contamination. Therefore, a shift toward faster decaying NMDAR-mediated currents could produce an apparent reduction in NMDA current. For example, a shift toward more GluN2A, which has faster decay kinetics, could produce less current even with more NMDARs (Lopez de and Sah, 2003; Gray et al., 2011; Paoletti et al., 2013). Alternatively, the diminished NMDA current could be caused by the lateral movement of NMDA receptors to the edges of the synapse which could be detected on the synaptosomes in spite of being less activated by the synaptic release of glutamate (Paoletti et al., 2013; Lussier et al., 2015). Another possibility is that both presynaptic and postsynaptic NMDA receptors were detected on the synaptosomes, but only postsynaptic receptors were assessed with the electrophysiology. However, we do not think that there were significant levels of presynaptic GluN1 in our sample, since the vast majority of GluN1 was found on the largest synaptosomes (Supplementary Figure 2A). It is also important to note that the synaptosomes include vHPC synapses onto both pyramidal and GABAergic neurons from all cortical areas, whereas the electrophysiological studies were restricted to vHPC synapses onto layer V pyramidal neurons of IL. Given the strong feedforward inhibition produced by vHPC projections to IL (Marek et al., 2018), it is possible that synapses onto GABAergic neurons contribute substantially to our results. Although the mechanism behind the previously observed reduction in NMDAR current remains to be determined, the analysis of GluN1 on vHPC-to-IL synaptosomes suggests that the reduction is not due to reduced expression of NMDARs.

## Conclusion

In summary, the use of ChR-EYFP combined with FACS allowed us to identify an increase in NMDARs specifically at the vHPC-to-IL synapse. This molecular change required the pairing of tones and shocks and occurred only in male rats. Our findings further indicate that fear acquisition alters IL physiology in male rats. The lack of similar synaptic plasticity in the females highlights the need to validate whether molecular changes identified in males also apply to females.

## Data Availability Statement

The raw data supporting the conclusions of this article will be made available by the authors, without undue reservation.

## Ethics Statement

The animal study was reviewed and approved by the institutional animal care and use committee.

## Author Contributions

YC-O, YG, and JP designed the research, analyzed the data, and wrote the manuscript. YC-O, MC, AH, and PL performed the research. All authors contributed to the article and approved the submitted version.

## Conflict of Interest

The authors declare that the research was conducted in the absence of any commercial or financial relationships that could be construed as a potential conflict of interest.

## Abbreviations

vHPC: ventral hippocampus
IL: infralimbic cortex
FACS: fluorescence-activated cell sorting
ANOVA: analysis of variance
NMDAR: *N*-methyl-D-aspartate receptor
PTSD: post-traumatic stress disorder.
