# Supplementary material for: Plasticity of GluN1 at Ventral Hippocampal Synapses in the Infralimbic Cortex
Source: Front Synaptic Neurosci. 2021 Jul 15;13:695964. doi: 10.3389/fnsyn.2021.695964 (PMC8320376; doi:10.3389/fnsyn.2021.695964)
Supplement: Supplementary file 1 [file Data_Sheet_1.PDF]

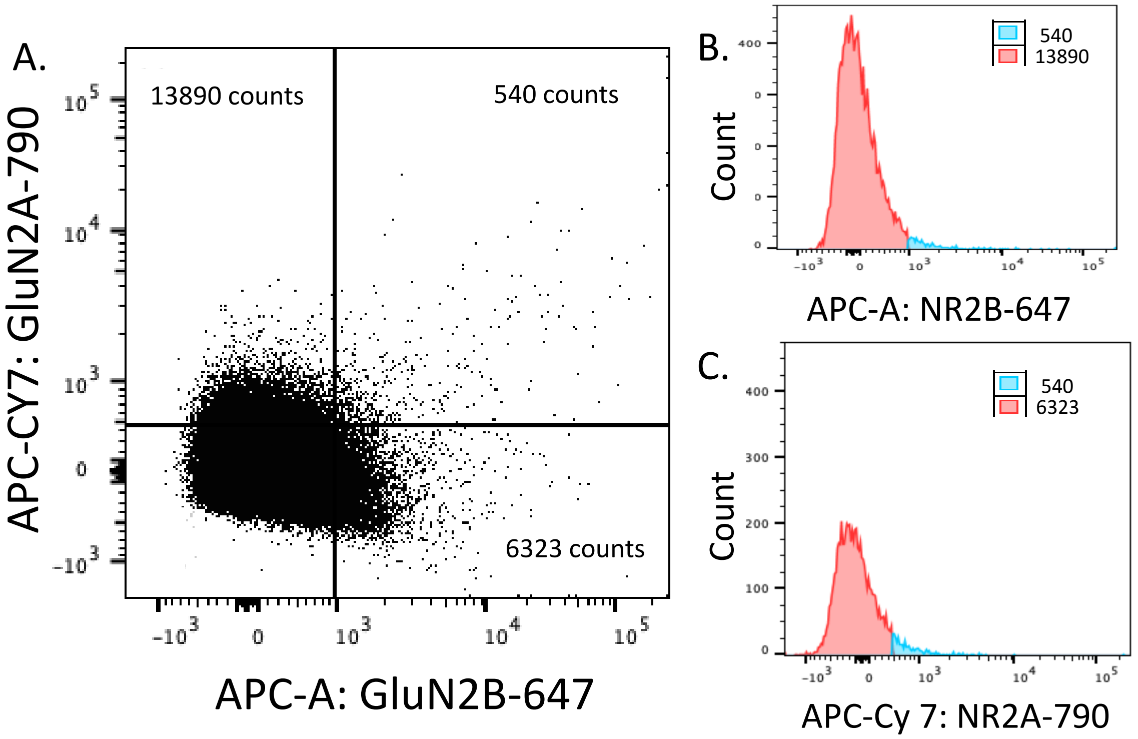


**Supplemental Figure 1: Aggregates of synaptosomes do not significantly contribute to the detection of double labeled synaptosomes.** A. Two synaptosome samples were separately labeled with antibodies conjugated to either Alexa Fluor 647 or Alexa Fluor 790 and then combined to determining the detection of double positive aggregates. B. & C. Histograms showing the detection of single positive synaptosomes (red) and double positive (blue, aggregates). Only 2.6% (540 synaptosomes) of the positive synaptosomes (20,753 synaptosomes) were double positive.

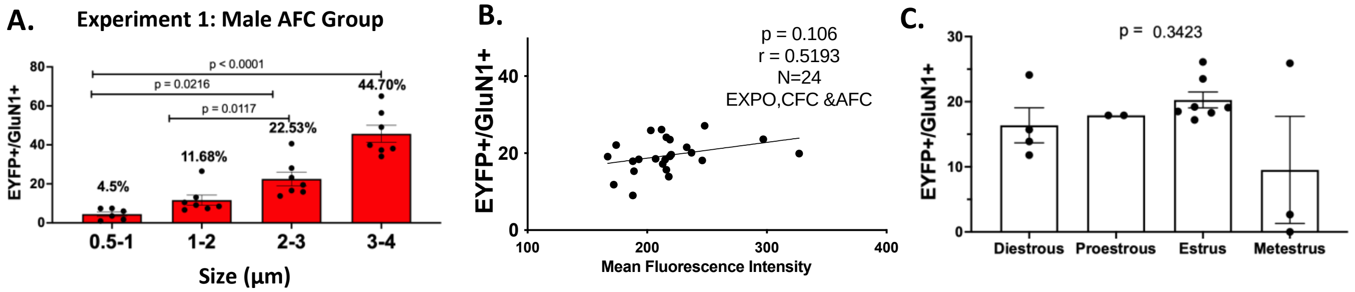


**Supplemental Figure 2:** A. Percent of EYFP+ vHPC-to-IL synaptosomes of different sizes (0.5-1, 1-2, 2-3, and 3-4 µm) that also express GluN1 in male AFC group. B. No correlation between EYFP+/GluN1+ and the mean fluorescence intensity in females from experiment 1. C. Percent of EYFP+/GluN1+ separated by stage of estrous cycle of female rats in experiment 1.

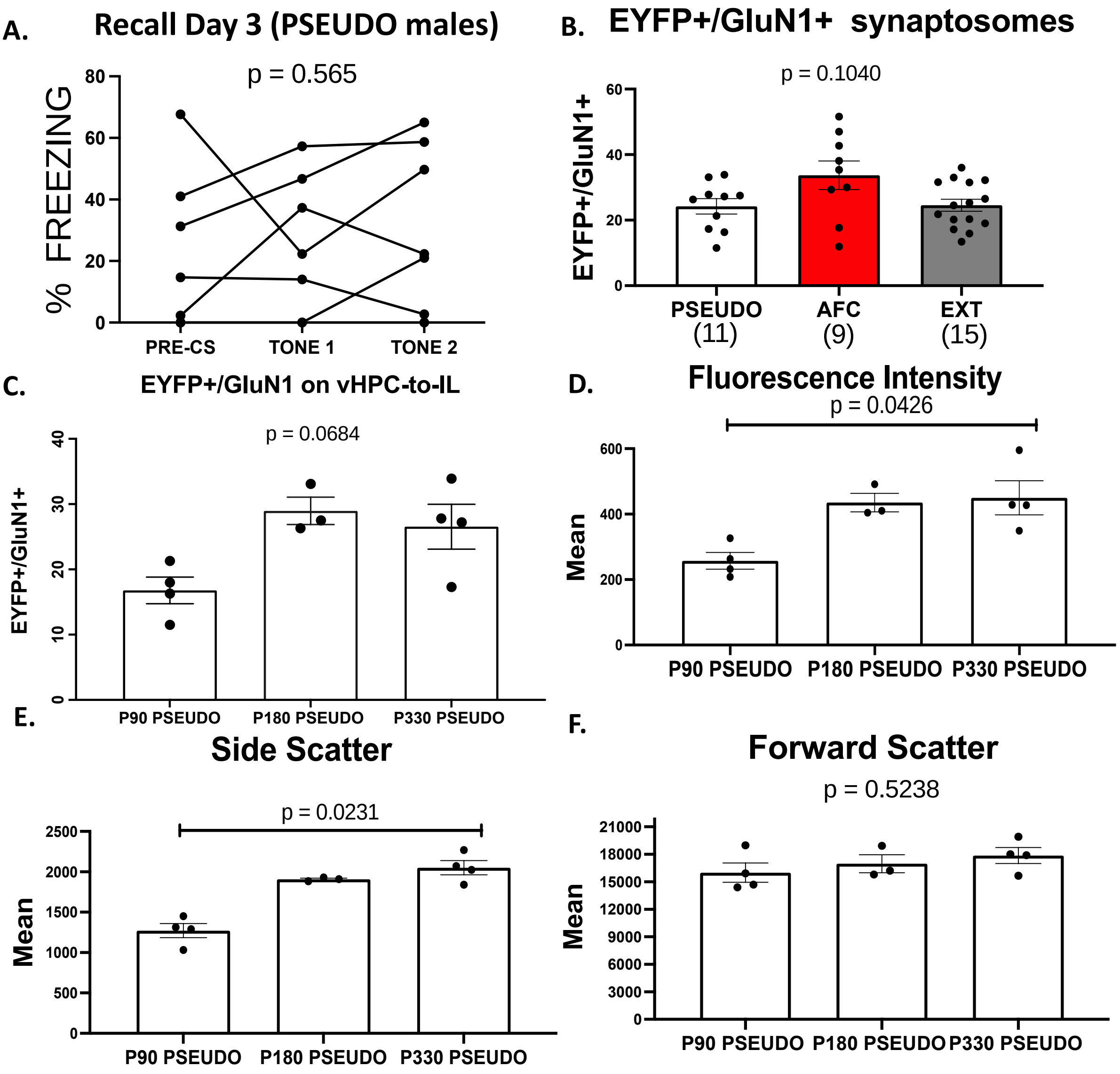


**Supplemental Figure 3: NMDARs on vHPC-to-IL synaptosomes in male rats change with age or time post-infection.** A. Percent of freezing before CS-tone and during each tone in male PSEUDO group at Recall Day 3. B. Levels of EYFP+/GluN1+ synaptosomes found in male groups without normalization by age. C. Levels of EYFP+/GluN1+ synaptosomes found in male PSEUDO groups of different ages. D-F. Mean fluorescence intensity of GluN1, side scatter, and forward scatter of EYFP+/GluN1+ synaptosomes in male PSEUDO groups of different ages.

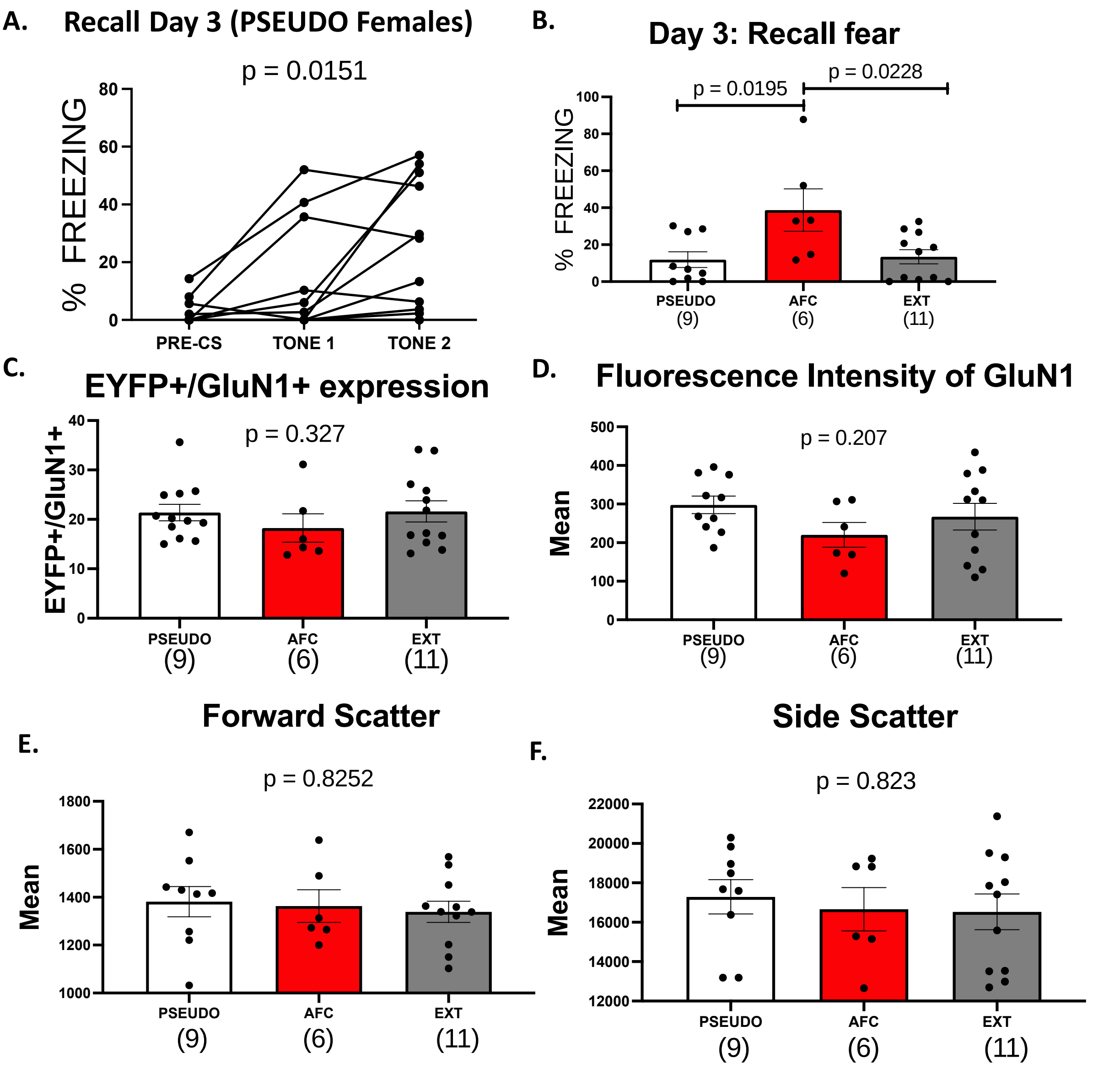


**Supplemental Figure 4: Removing high fear animals from PSUEDO and EXT groups does not reveal changes in NMDARs on vHPC-to-IL synaptosomes in female groups.** A. Percent of freezing before CS-tone and during each tone in female PSEUDO group at Recall Day 3. B. Fear recall in females after removing animals with more than 40% freezing from PSUEDO (3 animals) and EXT (1 animal) groups. C-F. Levels, mean fluorescence intensity of GluN1, forward scatter, and side scatter of EYFP+/GluN1+ synaptosomes in female groups after removing animals with more than 40% freezing from PSUEDO (3 animals) and EXT (1 animal) groups.

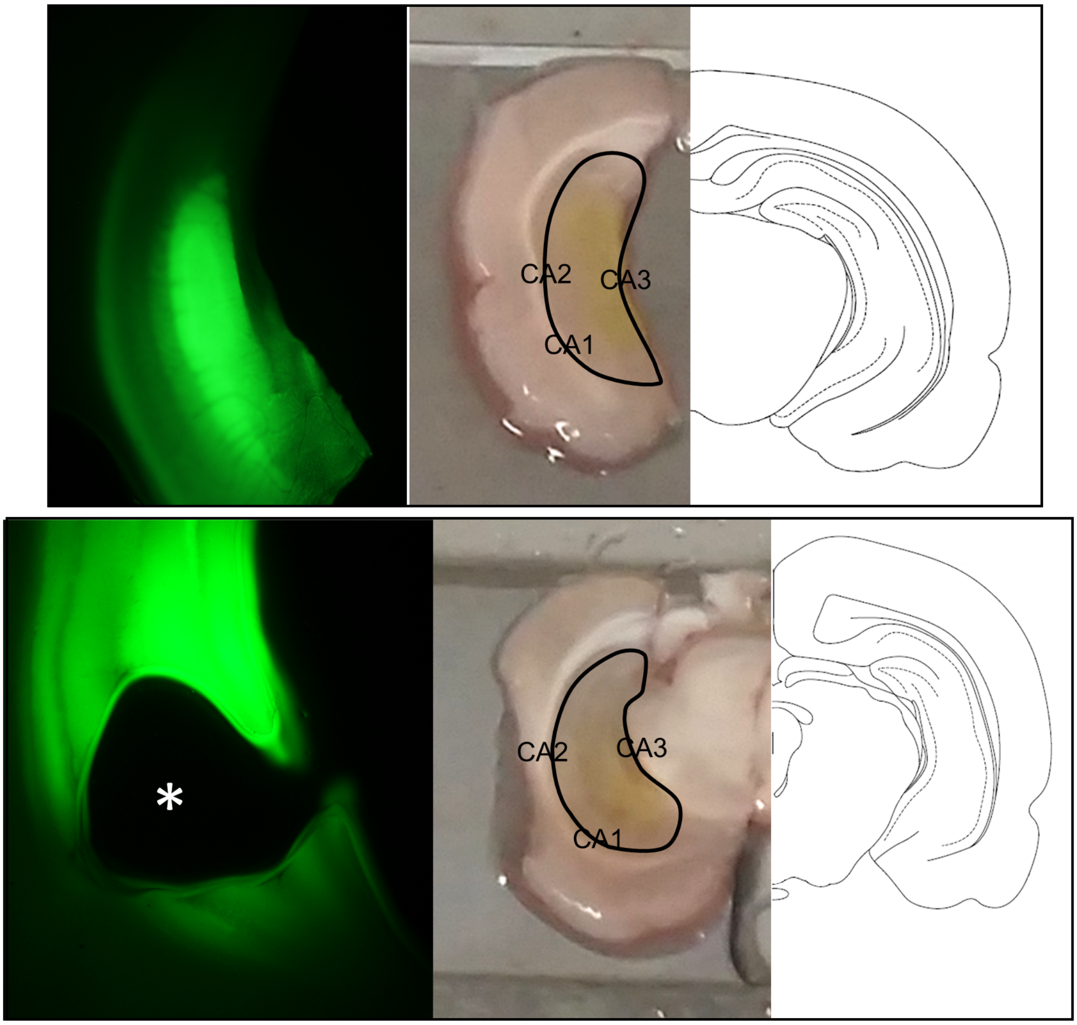


**Supplemental Figure 5:** On the left are epifluorescence images taken with Olympus BX60 microscope showing examples of EYFP expression in the vHPC. Astrix denotes where a tissue punch was taken from the vHPC for a different set of experiments. In the middle are brightfield images showing examples of fresh tissue.

Summary of statistical analyses of EYFP+/GluN1+ expression and  behavioral task.

| Figures | Measured condition | Groups analyzed | Factors | Analysis | |
|---|---|---|---|---|---|
| 1.A | Fear Expression: Percent of freezing | F: EXPOSURE,CFC and AFC | Normality and Lognormality test | Shapiro-Wilks | P Value |
| | | | | EXPOSURE | 0.004 |
| | | | | CFC | 0.926 |
| | | | | AFC | 0.971 |
| | | | ANOVA table | F (DFn, DFd) | P value |
| | | | Trials x Treatment | F (8, 88) = 3.408 | P=0.0019 |
| | | | Trials | F (2.794, 61.46) = 20.26 | P<0.0001 |
| | | | Treatment | F (2, 22) = 6.676 | P=0.0054 |
| | | | Animals | F (22, 88) = 7.588 | P<0.0001 |
| | | | TRIAL | | |
| | | | | Tukey's multiple comparisons test | |
| | | | | CFC | P Value |
| | | | | TRIAL 1 vs. TRIAL 2 | 0.0488 |
| | | | | TRIAL 1 vs. TRIAL 3 | <0.0001 |
| | | | | TRIAL 1 vs. TRIAL 4 | <0.0001 |
| | | | | TRIAL 1 vs. TRIAL 5 | <0.0001 |
| | | | | TRIAL 2 vs. TRIAL 3 | 0.0054 |
| | | | | TRIAL 2 vs. TRIAL 4 | 0.006 |
| | | | | TRIAL 2 vs. TRIAL 5 | 0.0112 |
| | | | | TRIAL 3 vs. TRIAL 4 | 0.5983 |
| | | | | TRIAL 3 vs. TRIAL 5 | 0.9909 |
| | | | | TRIAL 4 vs. TRIAL 5 | 0.6932 |
| | | | | EXPOSURE | |
| | | | | TRIAL 1 vs. TRIAL 2 | 0.2686 |
| | | | | TRIAL 1 vs. TRIAL 3 | 0.4056 |
| | | | | TRIAL 1 vs. TRIAL 4 | 0.0712 |
| | | | | TRIAL 1 vs. TRIAL 5 | 0.362 |
| | | | | TRIAL 2 vs. TRIAL 3 | 0.4965 |
| | | | | TRIAL 2 vs. TRIAL 4 | 0.9996 |
| | | | | TRIAL 2 vs. TRIAL 5 | 0.9997 |
| | | | | TRIAL 3 vs. TRIAL 4 | 0.2545 |
| | | | | TRIAL 3 vs. TRIAL 5 | 0.5333 |
| | | | | TRIAL 4 vs. TRIAL 5 | 0.9823 |
| | | | | AFC | |
| | | | | TRIAL 1 vs. TRIAL 2 | 0.0408 |
| | | | | TRIAL 1 vs. TRIAL 3 | 0.013 |
| | | | | TRIAL 1 vs. TRIAL 4 | 0.0021 |
| | | | | TRIAL 1 vs. TRIAL 5 | 0.0041 |
| | | | | TRIAL 2 vs. TRIAL 3 | 0.7331 |
| | | | | TRIAL 2 vs. TRIAL 4 | 0.2264 |
| | | | | TRIAL 2 vs. TRIAL 5 | 0.3532 |
| | | | | TRIAL 3 vs. TRIAL 4 | 0.4455 |
| | | | | TRIAL 3 vs. TRIAL 5 | 0.1781 |
| | | | | TRIAL 4 vs. TRIAL 5 | >0.9999 |
| | | | TREATMENT | | |
| | | | | EXPOSURE vs. CFC | <0.0001 |
| | | | | EXPOSURE vs. AFC | 0.0001 |
| | | | | CFC vs. AFC | 0.0018 |
| 1.B | Fear Expression: Percent of freezing | M: EXPOSURE,CFC and AFC | Normality and Lognormality test | Shapiro-Wilks | P Value |
| | | | | EXPOSURE | 0.47 |
| | | | | CFC | 0.76 |
| | | | | AFC | 0.41 |
| | | | ANOVA table | F (DFn, DFd) | P value |
| | | | Trials x Treatment | F (12, 80) = 10.98 | P<0.0001 |
| | | | Trials | F (2.087, 41.74) = 38.06 | P<0.0001 |
| | | | Treatment | F (3, 20) = 36.00 | P<0.0001 |
| | | | Animals | F (20, 80) = 3.663 | P<0.0001 |
| | | | TRIAL | | |
| | | | | Tukey's multiple comparisons test | |
| | | | | CFC | P Value |
| | | | | TRIAL 1 vs. TRIAL 2 | 0.1418 |
| | | | | TRIAL 1 vs. TRIAL 3 | 0.0869 |
| | | | | TRIAL 1 vs. TRIAL 4 | 0.0218 |
| | | | | TRIAL 1 vs. TRIAL 5 | 0.0165 |
| | | | | TRIAL 2 vs. TRIAL 3 | 0.8774 |
| | | | | TRIAL 2 vs. TRIAL 4 | 0.3581 |
| | | | | TRIAL 2 vs. TRIAL 5 | 0.1413 |
| | | | | TRIAL 3 vs. TRIAL 4 | 0.7057 |
| | | | | TRIAL 3 vs. TRIAL 5 | 0.5423 |
| | | | | TRIAL 4 vs. TRIAL 5 | 0.4908 |
| | | | | EXPOSURE | |
| | | | | TRIAL 1 vs. TRIAL 2 | 0.6223 |
| | | | | TRIAL 1 vs. TRIAL 3 | 0.1662 |
| | | | | TRIAL 1 vs. TRIAL 4 | 0.5985 |
| | | | | TRIAL 1 vs. TRIAL 5 | 0.2485 |
| | | | | TRIAL 2 vs. TRIAL 3 | 0.8847 |
| | | | | TRIAL 2 vs. TRIAL 4 | 0.8771 |
| | | | | TRIAL 2 vs. TRIAL 5 | 0.3535 |
| | | | | TRIAL 3 vs. TRIAL 4 | >0.9999 |
| | | | | TRIAL 3 vs. TRIAL 5 | 0.8364 |
| | | | | TRIAL 4 vs. TRIAL 5 | 0.7695 |
| | | | | AFC | |
| | | | | TRIAL 1 vs. TRIAL 2 | 0.8469 |
| | | | | TRIAL 1 vs. TRIAL 3 | 0.4934 |
| | | | | TRIAL 1 vs. TRIAL 4 | 0.0037 |
| | | | | TRIAL 1 vs. TRIAL 5 | 0.0008 |
| | | | | TRIAL 2 vs. TRIAL 3 | 0.5325 |
| | | | | TRIAL 2 vs. TRIAL 4 | 0.0917 |
| | | | | TRIAL 2 vs. TRIAL 5 | 0.138 |
| | | | | TRIAL 3 vs. TRIAL 4 | 0.1183 |
| | | | | TRIAL 3 vs. TRIAL 5 | 0.2509 |
| | | | | TRIAL 4 vs. TRIAL 5 | 0.9748 |
| | | | TREATMENT | | |
| | | | | Tukey's multiple | P value |
| | | | | EXPOSURE vs. CFC | 0.003 |
| | | | | EXPOSURE vs. AFC | 0.004 |
| | | | | CFC vs. AFC | 0.6143 |
| 1.C | Day 2: Fear Recall | F:EXPOSURE,CFC and AFC | ANOVA | F (DFn, DFd) | P value |
| | | | FEMALES | 3.954 (2, 22) | p = 0.034 |
| | | | | Tukey's multiple comparisons test | |
| | | | TREATMENT | EXPO vs CFC | 0.0009 |
| | | | | EXPO vs AFC | 0.0157 |
| | | | | CFC vs AFC | 0.629 |
| 1.D | Day 2: Fear Recall | M: EXPOSURE,CFC and AFC | ANOVA | F (DFn, DFd) | P value |
| | | | MALES | 3.608 (2, 21) | 0.045 |
| | | | | Tukey's multiple comparisons test | |
| | | | TREATMENT | EXPO vs CFC | 0.0003 |
| | | | | EXPO vs AFC | 0.0029 |
| | | | | CFC vs AFC | 0.912 |
| 3.A | EYFP+/GluN1+ | EXPOSURE,CFC and AFC | Treatment M | ANOVA | p = 0.01 |
| | | | | Dunn's multiple comparisons test | |
| | | | | EXPO vs. CFC | >0.9999 |
| | | | | EXPO vs AFC | p = 0.023 |
| | | | | CFC vs AFC | p = 0.021 |
| 3.C | Mean Fluorescence Intensity of EYFP+/GluN1+ | M: EXPOSURE,CFC and AFC | Treatment | Shapiro Wilks | P Value |
| | | | | N=25 | 0.0005 |
| | | | ANOVA | F (DFn, DFd) | P value |
| | | | | H (2, 22) = 9.624 | p = 0.0081 |
| | | | | Dunn's multiple comparisons test | P Value |
| | | | | EXPOSURE vs. CFC | >0.9999 |
| | | | | EXPOSURE vs. AFC | 0.0283 |
| | | | | CFC vs. AFC | 0.0125 |
| 3.C | Correlation: GluN1 vs Fluorescence Intensity | M:  EXPOSURE,CFC and AFC | MALES | P value | 0.007 |
| | | | N-= 25 | r | 0.5193 |
| 3.E | Mean Forward Scatter of EYFP+/GluN1+ | M: EXPOSURE,CFC and AFC | Treatment | Shapiro Wilks | P Value |
| | | | | N=25 | 0.11 |
| | | | ANOVA | F (DFn, DFd) | P value |
| | | | | H (2, 22) = 3.67 | p = 0.1594 |
| | | | | Dunn's multiple comparisons test | P Value |
| | | | | EXPOSURE vs. CFC | >0.9999 |
| | | | | EXPOSURE vs. AFC | 0.1679 |
| | | | | CFC vs. AFC | 0.6997 |
| 3.E | Mean Side Scatter of EYFP+/GluN1+ | M: EXPOSURE,CFC and AFC | Treatment | Shapiro Wilks | P Value |
| | | | | N=25 | 0.34 |
| | | | ANOVA | F (DFn, DFd) | P value |
| | | | | H (2, 22 ) = 9.485 | p = 0.008 |
| | | | | Dunn's multiple comparisons test | P Value |
| | | | | EXPOSURE vs. CFC | >0.9999 |
| | | | | EXPOSURE vs. AFC | 0.0342 |
| | | | | CFC vs. AFC | 0.0121 |
| 4.A | EYFP+/GluN1+ | EXPOSURE,CFC and AFC | Normality and Lognormality test | Shapiro-Wilks | P Value |
| | | | | N=59 | <0.0001 |
| | | | ANOVA | F (DFn, DFd) | P value |
| | | | Females | H (2, 21) = 1.609 | p = 0.44 |
| | | | Males | H (2, 22) = 9.166 | p = 0.01 |
| | | | | Dunn's multiple comparisons test | Adjusted P Value |
| | | | | EXPO vs. CFC | >0.9999 |
| | | | | EXPO vs. AFC | 0.7348 |
| | | | | CFC vs. AFC | 0.8099 |
| 4.C | Mean Fluorescence Intensity of EYFP+/GluN1+ | F: EXPOSURE,CFC and AFC | Treatment | ANOVA | p = 0.01 |
| | | | | Dunn's multiple comparisons test | |
| | | | ANOVA | EXPO vs. CFC | >0.9999 |
| | | | | EXPO vs AFC | p = 0.023 |
| | | | | CFC vs AFC | p = 0.0208 |
| 4.D | EYFP+/GluN1+ at stages of estrous cycle | F: Exposure, CFC and AFC | Females: Estrous Cycle | | P value |
| | | | N-= 17 | | 0.7374 |
| 4.E | Mean Forward Scatter of EYFP+/GluN1+ | F: EXPOSURE,CFC and AFC | Treatment | | P Value |
| | | | ANOVA | | 0.9403 |
| 4.F | Mean Side Scatter of EYFP+/GluN1+ | F: EXPOSURE,CFC and AFC | Treatment | ANOVA | P-VALUES |
| | | | | 0.0197 | |
| | | | ANOVA | Dunn's multiple comparisons test | |
| | | | | EXPO vs. CFC | >0.9999 |
| | | | | EXPO vs. AFC | 0.0387 |
| | | | | CFC vs. AFC | 0.037 |
| 5.A | Fear Expression: Percent of freezing | F: PSEUDO, AFC and EXT | ANOVA table | F (DFn, DFd) | P value |
| | | | Trials | F (33, 10) = 1.187 | P=0.4074 |
| | | | Treatment | F (2, 10) = 7.554 | P=0.0100 |
| | | | | Tukey's multiple comparisons test | Adjusted P Value |
| | | | | HAB | |
| | | | | EXTINCTION vs. AFC | 0.9447 |
| | | | | EXTINCTION vs. PSEUDO | 0.9796 |
| | | | | AFC vs. PSEUDO | 0.8793 |
| | | | | COND 1 | |
| | | | | EXTINCTION vs. AFC | 0.7406 |
| | | | | EXTINCTION vs. PSEUDO | 0.5459 |
| | | | | AFC vs. PSEUDO | 0.9851 |
| | | | | COND 2 | |
| | | | | EXTINCTION vs. AFC | 0.9951 |
| | | | | EXTINCTION vs. PSEUDO | 0.0201 |
| | | | | AFC vs. PSEUDO | 0.2354 |
| | | | | COND3 | |
| | | | | EXTINCTION vs. AFC | 0.9909 |
| | | | | EXTINCTION vs. PSEUDO | <0.0001 |
| | | | | AFC vs. PSEUDO | 0.0128 |
| | | | | COND 4 | |
| | | | | EXTINCTION vs. AFC | 0.9995 |
| | | | | EXTINCTION vs. PSEUDO | <0.0001 |
| | | | | AFC vs. PSEUDO | 0.0018 |
| | | | | COND 5 | |
| | | | | EXTINCTION vs. AFC | 0.7016 |
| | | | | EXTINCTION vs. PSEUDO | 0.0001 |
| | | | | AFC vs. PSEUDO | 0.091 |
| 5.B | Fear Expression: Percent of freezing | M: PSEUDO, AFC and EXT | ANOVA table | F (DFn, DFd) | P value |
| | | | Trials | F (33, 10) = 1.585 | P=0.2233 |
| | | | Treatment | F (2, 10) = 9.666 | P=0.0046 |
| | | | | Tukey's multiple comparisons test | Adjusted P Value |
| | | | | HAB | |
| | | | | EXTINCTION vs. AFC | 0.924 |
| | | | | EXTINCTION vs. PSEUDO | 0.9718 |
| | | | | AFC vs. PSEUDO | 0.8259 |
| | | | | COND 1 | |
| | | | | EXTINCTION vs. AFC | 0.9975 |
| | | | | EXTINCTION vs. PSEUDO | 0.9566 |
| | | | | AFC vs. PSEUDO | 0.9805 |
| | | | | COND 2 | |
| | | | | EXTINCTION vs. AFC | 0.9991 |
| | | | | EXTINCTION vs. PSEUDO | 0.0008 |
| | | | | AFC vs. PSEUDO | 0.0263 |
| | | | | COND3 | |
| | | | | EXTINCTION vs. AFC | 0.8117 |
| | | | | EXTINCTION vs. PSEUDO | 0.0008 |
| | | | | AFC vs. PSEUDO | 0.0143 |
| | | | | COND 4 | |
| | | | | EXTINCTION vs. AFC | 0.6117 |
| | | | | EXTINCTION vs. PSEUDO | 0.0006 |
| | | | | AFC vs. PSEUDO | 0.0001 |
| | | | | COND 5 | |
| | | | | EXTINCTION vs. AFC | 0.9993 |
| | | | | EXTINCTION vs. PSEUDO | 0.0168 |
| | | | | AFC vs. PSEUDO | 0.0269 |
| 6.A | Fear Expression: Percent of freezing | M: PSEUDO, AFC, EXT | ANOVA | F (DFn, DFd) | P value |
| | | | | F (2, 32) = 2.217 | p = 0.0013 |
| | | | | Tukey's multiple comparisons test | |
| | | | | PSEUDO vs. AFC | 0.1001 |
| | | | | PSEUDO vs. EXT | 0.1845 |
| | | | | AFC vs. EXT | 0.0009 |
| 6.B | EYFP+/GluN1+ | M: PSEUDO, AFC, EXT | Normality and Lognormality test | Shapiro-Wilks | |
| | | | | N=59 | <0.0001 |
| | | | ANOVA | F (DFn, DFd) | |
| | | | | H (2, 32) = 7.182 | p = 0.028 |
| | | | | Dunn's multiple comparisons test | |
| | | | | PSEUDO vs. AFC | 0.0382 |
| | | | | PSEUDO vs. EXT | 0.3241 |
| | | | | AFC vs. EXT | 0.7393 |
| 6.C | Mean Fluorescence Intensity of EYFP+/GluN1+ | M: PSEUDO, AFC, EXT | ANOVA | F (DFn, DFd) | |
| | | | | H (2, 32) = 6.201 | p = 0.045 |
| | | | Treatment | Dunn's multiple comparisons test | |
| | | | | PSEUDO vs. AFC | 0.0304 |
| | | | | PSEUDO vs. EXT | 0.156 |
| | | | | AFC vs. EXT | >0.9999 |
| 6.D | Mean Side Scatter of EYFP+/GluN1+ | M: PSEUDO, AFC, EXT | ANOVA | F (DFn, DFd) | |
| | | | | H (2, 32) = 1.88 | p = 0.39 |
| | | | Treatment | ANOVA | |
| | | | | | 0.6 |
| 6.E | Mean Forward Scatter of EYFP+/GluN1+ | M: PSEUDO, AFC, EXT | ANOVA | F (DFn, DFd) | |
| | | | | H (2, 32) = 0.28 | p = 0.87 |
| | | | Treatment | ANOVA | |
| | | | | | 0.5 |
| 7.A | Fear Expression: Percent of freezing | F: PSEUDO, AFC, EXT | ANOVA | F (DFn, DFd) | |
| | | | | F (2, 27) = 0.487 | p = 0.144 |
| | | | | Tukey's multiple comparisons test | |
| | | | | PSEUDO vs. AFC | 0.259 |
| | | | | PSEUDO vs. EXT | 0.8768 |
| | | | | AFC vs. EXT | 0.1293 |
| 7.B | EYFP+/GluN1+ | F: PSEUDO, AFC, EXT | ANOVA | F (DFn, DFd) | |
| | | | | H (2, 27) = 2.274 | p = 0.3207 |
| | | | Treatment | ANOVA | |
| | | | | | 0.3 |
| 7.C | Mean Fluorescence Intensity of EYFP+/GluN1+ | F: PSEUDO, AFC, EXT | ANOVA | F (DFn, DFd) | |
| | | | | H (2, 27) = 4,63 | p = 0.098 |
| | | | Treatment | ANOVA | |
| | | | | | 0.0983 |
| 7.D | Mean Forward Scatter of EYFP+/GluN1+ | F: PSEUDO, AFC, EXT | ANOVA | F (DFn, DFd) | |
| | | | | H (2, 27) = 0.667 | |
| | | | Treatment | ANOVA | p = 0.7154 |
| 7.E | Mean Side Scatter of EYFP+/GluN1+ | F: PSEUDO, AFC, EXT | ANOVA | F (DFn, DFd) | |
| | | | | H (2, 27) = 0.61 | |
| | | | Treatment | ANOVA | p = 0.7372 |

SUPPLEMENTARY
